# Coronary Artery Vasospasm After Mechanical Aortic Valve Replacement: A Case Report

**DOI:** 10.7759/cureus.49747

**Published:** 2023-11-30

**Authors:** Elen Mussie, Natalie DeWitte, Michael Goulet, Jamie Garfield

**Affiliations:** 1 Department of Radiology, Lewis Katz School of Medicine at Temple University, Temple University Hospital, Philadelphia, USA; 2 Department of General Surgery, Lewis Katz School of Medicine at Temple University, Temple University Hospital, Philadelphia, USA; 3 Department of Thoracic Medicine and Surgery, Lewis Katz School of Medicine at Temple University, Temple University Hospital, Philadelphia, USA

**Keywords:** diffuse coronary artery spasm, intracoronary nitroglycerin, extracorporeal membrane oxygenation, aortic valve replacement, coronary vasospasm

## Abstract

Coronary artery vasospasm is a rare but fatal postoperative complication of cardiothoracic surgery. This phenomenon can occur directly after surgery or several hours postoperatively. Most reported cases have occurred after CABG surgery and less commonly after valve replacement. Patients can present with various symptoms, and physicians must be familiar with the indications to suspect coronary artery vasospasm to avoid adverse outcomes. We present a case of a 60-year-old female who suffered a cardiac arrest with refractory ventricular fibrillation due to coronary artery vasospasm following aortic valve replacement. During resuscitation, she underwent central veno-arterial extracorporeal membrane oxygenation (VA-ECMO) cannulation for hemodynamic support. She subsequently underwent urgent left heart catheterization, revealing vasospasm of the left anterior descending artery, early first diagonal, early first obtuse marginal, and non-dominant right coronary artery. Vasospasm was successfully treated with intracoronary nitroglycerin and nicardipine. This case report demonstrates the importance of early consideration of coronary artery vasospasm as a cause of postoperative arrest following cardiac surgery.

## Introduction

Coronary artery vasospasm is the near complete or complete occlusion of the vessel secondary to constriction. This process can cause ischemia, result in acute coronary syndrome, and be fatal if not promptly recognized and treated [1]. Coronary artery vasospasm is a rare outcome of cardiothoracic surgery despite the high rate of procedures in the United States. It has been reported as a complication in approximately 0.43-1.3% of CABG procedures. This complication is not well understood, and delays in diagnosis are fatal [2]. Patients can be asymptomatic or present with hemodynamic instability, circulatory collapse, and ischemic-like ECG changes [3]. Rapid diagnosis is necessary with coronary angiography to avoid mortality. Treatment options include intracoronary vasodilators and adjuvant hemodynamic support with extracorporeal membrane oxygenation (ECMO), which is necessary to avoid circulatory collapse [2]. We present a case of a patient developing coronary artery vasospasm after aortic valve replacement.

## Case presentation

A 60-year-old female with a history of heart failure with preserved ejection fraction (EF), multiple sclerosis, severe aortic insufficiency, and atrial flutter underwent mechanical aortic valve replacement, cryoablation for cavo-tricuspid isthmus, and left atrial appendage clipping on cardiopulmonary bypass. Her pre-operative workup included a transesophageal echocardiogram (TEE), which demonstrated a mildly dilated left ventricle with an estimated EF of 60-65% and moderate-to-severe aortic transvalvular regurgitation. Pre-operative ECG showed typical atrial flutter and a left cardiac catheterization showed no significant coronary artery disease. 

After surgery, intraoperative TEE showed an estimated EF of 55-60% with no further evidence of aortic regurgitation. She returned from the operating room to the intensive care unit intubated, on epinephrine, norepinephrine, and vasopressin for hemodynamic support. Vasopressors were weaned to low-dose vasopressin and epinephrine. She was extubated on postoperative day one. Three hours post-extubation, she developed dyspnea, vomiting, pre-syncope, and hypotension. She became unresponsive and went into pulseless electrical activity arrest.

**Figure 1:**
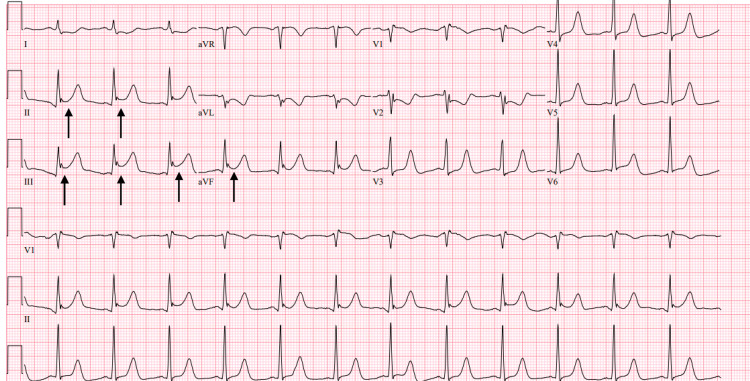
ECG with inferior STEMI. Arrows correlating to ST elevations in leads II, III, and aVF.

She underwent one round of ACLS before ROSC. At that time, ECG revealed new ST elevations in inferior leads concerning ischemic injury caused by an occlusion of the right coronary artery (Figure 1). An echocardiogram revealed a new biventricular failure with a small posterior pericardial effusion. STEMI-alert was called, but the patient had cardiopulmonary arrest again within minutes. 

**Figure 2:**
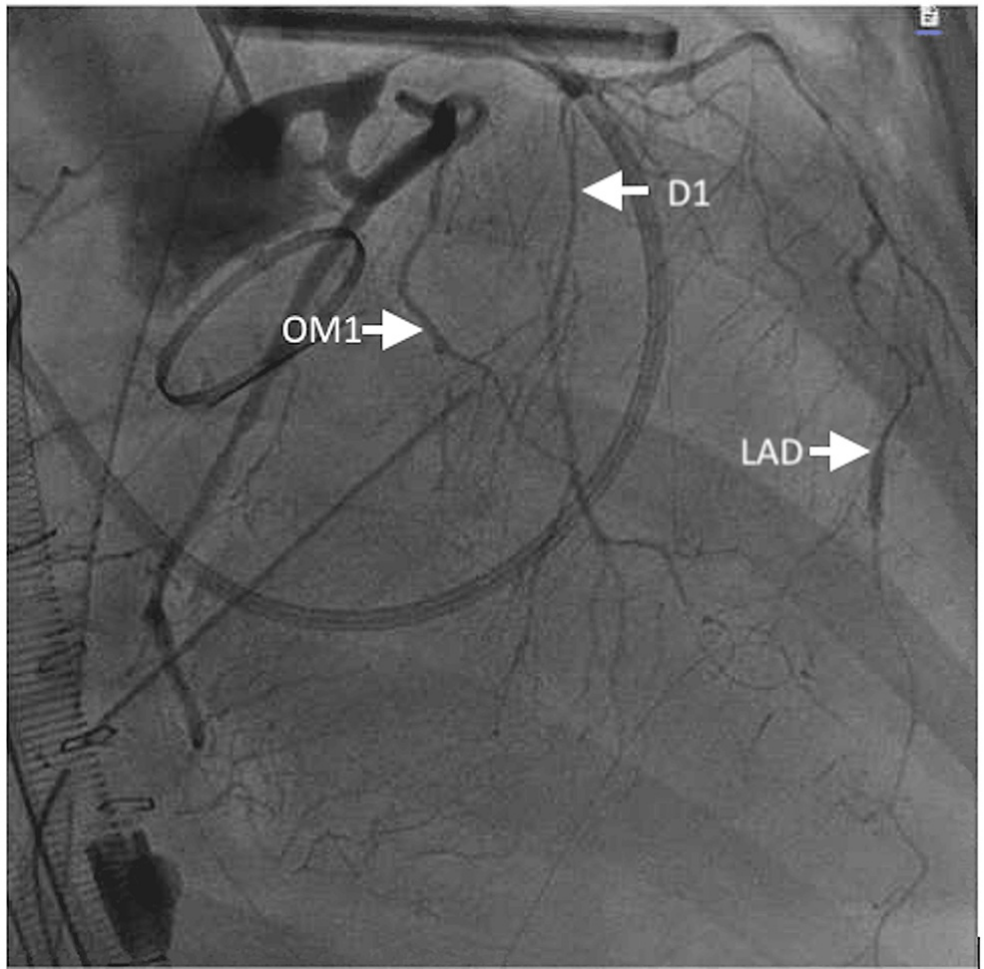
Coronary angiogram. RAO caudal view of left dominant coronary artery system. Note diffuse spasm along the entire left anterior descending, early first diagonal, and first obtuse marginal arteries with relative sparing of left main coronary artery and left circumflex LAD, left anterior descending artery; OM1, first obtuse marginal artery; D1, first diagonal artery

**Figure 3:**
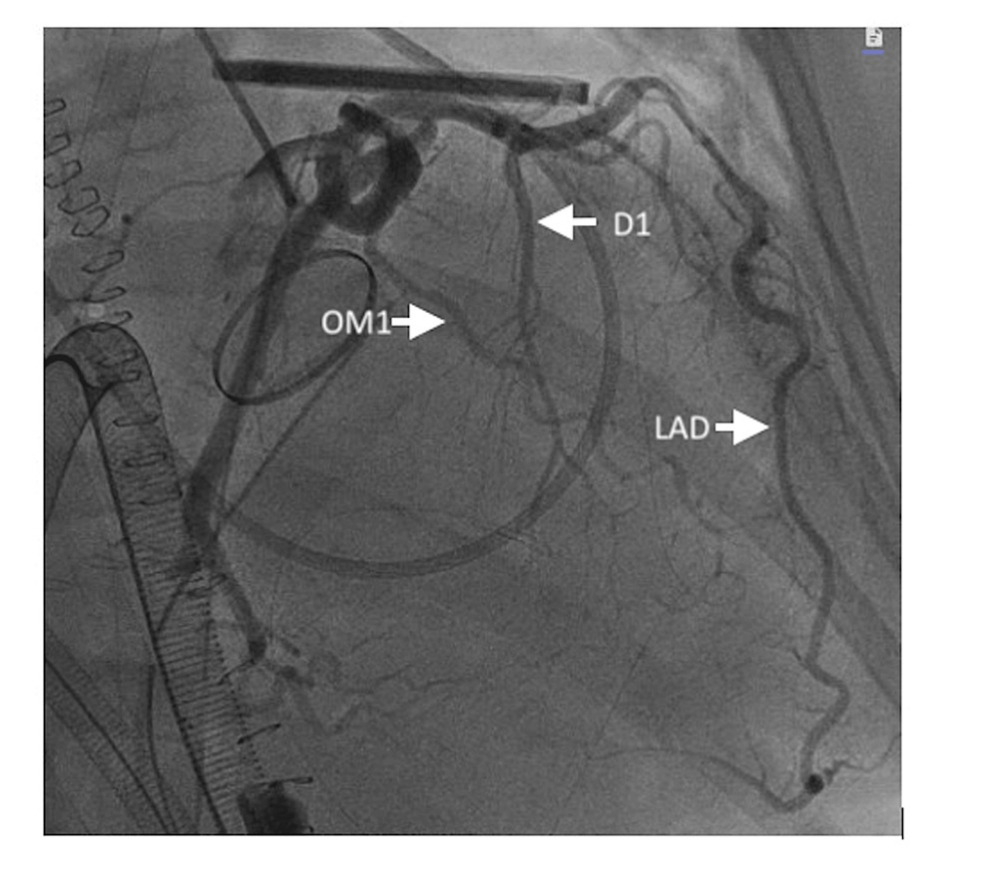
Coronary angiogram following intracoronary injections of nitroglycerin and nicardipine. RAO caudal view demonstrates recovery of vessel patency in the left anterior descending, first diagonal branch, and first obtuse marginal arteries LAD, left anterior descending artery; OM1, first obtuse marginal artery; D1, first diagonal artery

She developed pulseless ventricular fibrillation refractory to external defibrillation and chemical cardioversion. The chest was re-opened for cardiac massage and manual internal defibrillation. She was subsequently cannulated for central veno-arterial (VA)-ECMO. A minor laceration on the right ventricle was identified and repaired. The laceration was most likely caused by trauma from chest compressions. She was resuscitated with blood and intravenous crystalloid. Upon ROSC, she was taken to the operating room for chest re-exploration. She underwent left heart catheterization, demonstrating severe spasm of the left anterior descending artery, early first diagonal, early first obtuse marginal, and non-dominant right coronary artery (Figure 2, Video 1). The diffuse spasm was resolved with intracoronary nitroglycerin and nicardipine (Figure 3). TEE revealed severely reduced left ventricular function with an estimated EF of 10-15%, severely reduced right ventricular function, and appropriate motion of both aortic valve leaflets. 

**Video 1:** RAO caudal shot demonstrating severe coronary vasospasm in a left dominant system, most notably in the left anterior descending artery, first diagonal branch, and first obtuse marginal branch

She was maintained on VA-ECMO for hemodynamic support. She was managed with intravenous diltiazem, nicardipine, and nitroglycerin to prevent recurrence of coronary artery vasospasm. She was simultaneously placed on a low-dose epinephrine infusion for inotropic support in order to maintain pulsatility through the aortic valve and prevent left ventricular thrombus formation. 

Diltiazem infusion was discontinued on postoperative day three. She returned to the operating room on postoperative day four for VA-ECMO decannulation, chest washout, and closure. Nitroglycerin infusion was then slowly weaned off and discontinued on postoperative day 15, followed by the nicardipine infusion on postoperative day 18. Infusions were replaced with oral diltiazem and oral isosorbide dinitrate. Four days after ECMO decannulation, TEE revealed normal left and right ventricular size and systolic function. The estimated EF was 55-60% and the bi-leaflet mechanical aortic valve was well-seated. 

She underwent tracheostomy placement for prolonged ventilator-dependent respiratory failure and was discharged to rehab on hospital day 25. 

## Discussion

Coronary artery vasospasm is an extreme form of vasoconstriction affecting the arterial blood vessels [2]. Coronary artery vasospasm is a rare and often fatal complication of cardiac surgery. Cases of coronary vasospasm are more commonly reported after CABG and less commonly after valve surgery. The incidence rate of coronary artery vasospasm after CABG is reported to range between 0.43% and 1.3%. However, the incidence is likely underreported due to a low index of suspicion and inability to diagnose without coronary angiography [2]. Typically, the balance of endogenous vasoconstrictor and vasodilator mediators prevents the occurrence of vasospasm. The mechanism that disrupts this equilibrium remains unknown [2]. Several precipitating factors have been proposed, including increased catecholamine levels, physical manipulation of coronary arteries, platelet release of vasoconstrictive mediators, respiratory alkalosis, hypothermia, and sympatho-adrenergic stimulation [3,4]. In this case, the patient was extubated a few hours before the onset of coronary vasospasm. We hypothesize that endotracheal extubation caused a catecholamine release, prompting coronary artery vasoconstriction by stimulating alpha- and beta-adrenergic receptors [5]. 

Most reported cases of coronary vasospasm occur in the immediate postoperative period, ranging from three to eight hours after surgery [2]. The clinical presentation can range from asymptomatic ischemic ST changes and new regional wall abnormalities to hemodynamic instability and circulatory collapse [2,3]. The most dangerous type of coronary vasospasm is diffuse vasospasm, which involves the entire coronary artery system [6]. Patients often deteriorate quickly in these cases due to cardiogenic shock and fatal arrhythmias [2]. 

There are no consensus guidelines for the management of coronary vasospasm. However, direct injection of intracoronary vasodilators, including nitrates and calcium channel blockers, has been shown to improve blood flow and resolve coronary artery spasms [3,4]. Circulatory support with ECMO is often necessary before initiating intracoronary vasodilators. Without hemodynamic support of ECMO in cases of diffuse coronary vasospasm, rapid circulatory collapse can result in death prior to undergoing angiography [6]. 

Our patient’s presentation included hemodynamic instability and ventricular fibrillation refractory to defibrillation and chemical cardioversion. Identifying an acute reduction in EF on echocardiogram and ECG findings consistent with inferior STEMI prompted her to undergo coronary angiography despite her recent pre-operative catheterization showing no coronary artery disease. Our patient was promptly cannulated on VA-ECMO after recognizing the need for additional hemodynamic support. The administration of intracoronary nitroglycerin and nicardipine effectively resolved the coronary vasospasm. After undergoing angiography, our patient remained in the cardiothoracic intensive care unit on continuous intravenous nitrate until postoperative day 15, diltiazem until postoperative day three, and nicardipine on postoperative day 18. She had no further episodes of coronary vasospasm, her neurocognitive function remained intact, and her cardiac function recovered. She was discharged on 60 mg oral diltiazem and 40 mg oral isosorbide dinitrate. 

## Conclusions

In summary, we report a case of diffuse coronary artery vasospasm and resultant ventricular fibrillation following aortic valve replacement. The patient required emergent re-opening of the chest, manual internal defibrillation, and central VA-ECMO cannulation. Echocardiogram and ECG demonstrated new biventricular failure and STEMI, leading to urgent left heart catheterization. Vasospasm was quickly diagnosed and successfully treated. The patient survived hospital discharge with neurocognitive function intact. Several case reports describe vasospasm following CABG but fewer following valve surgery. This case report demonstrates how early detection of coronary artery vasospasm can dictate the patient's clinical course. Following cardiac surgery, patients should be closely monitored in an intensive care unit and providers should have a high index of suspicion for coronary vasospasm in the event of hemodynamic collapse. Due to its rare occurrence, symptom variability, and unpredictable clinical course, coronary artery vasospasm is challenging to recognize but can be successfully treated with early detection. 
